# Correction: A randomised survey of the quality of antibiotics and other essential medicines in Indonesia, with volume-adjusted estimates of the prevalence of substandard medicines

**DOI:** 10.1371/journal.pgph.0004740

**Published:** 2025-05-27

**Authors:** Elizabeth Pisani, Ayu Rahmawati, Esti Mulatsari, Mawaddati Rahmi, William Nathanial, Yusi Anggriani, on behalf of the STARmeds Study Group

The labels in one data pairing in Fig 3 are reversed. The authors have provided a corrected version. Please see the correct Fig 3 below.

**Fig 3:**
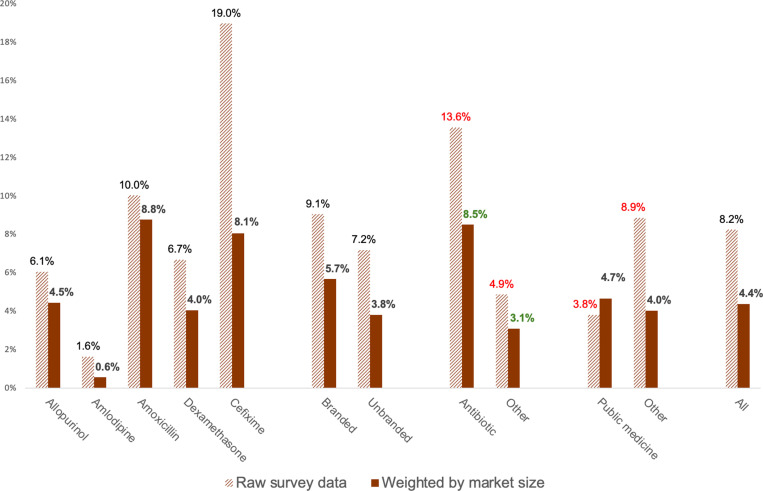
Raw and adjusted prevalence for study medicines. Red value labels denote statistically significant differences in analysis of raw data; green labels note differences significant in the adjusted analysis.
