# Supplementary material for: A randomised survey of the quality of antibiotics and other essential medicines in Indonesia, with volume-adjusted estimates of the prevalence of substandard medicines
Source: PLOS Glob Public Health. 2024 Dec 12;4(12):e0003999. doi: 10.1371/journal.pgph.0003999 (PMC11637375; doi:10.1371/journal.pgph.0003999)
Supplement: Attachment — Submitted filename: response_to_reviewers_1209.docx [file pgph.0003999.s003.docx]

Reviewer #1: This is an important article about prevalence of substandard medications in middle and low income countries, with a focus on Indonesia. It has a good design. I recommend it for publication and make a few suggestions for improving the article.

*Thank you.*

The abstract lacks a conclusion and summary at the end. Suggest adding a clear conclusion for lay audiences.

Similarly, the conclusion isn't very conclusive. I suggest the authors look at it again with fresh eyes and explain to lay readers the prevalence of the substandard medicines and their interpretation. It's unclear whether they think this is a problem of public health importance or not, and what the total prevalence of substandard medicines is. I think they are suggesting this isn't a big public health problem, but I read it twice and i'm still confused.

*Thanks for highlighting what was a dilemma for the authors. Our study suggests that the problem is not as much of a threat to public health as some of the more hyperbolic editorials of late have suggested, and that even academically rigorous meta-analyses probably overstate prevalence. However, as we mention in lines 79 and 93 of the original manuscript, Indonesia differs from many other middle and virtually all low income countries in that it has a relatively well-resourced regulator which oversees most essential medicines from production to use (since imports of off-patent essential medicines are rare). We are thus reluctant to draw conclusions that can not reliably be extrapolated globally. Having said that, we did note (lines 337-340 of the original manuscript) that the particular factors which led to over-estimates of the threat in our study are also present in other studies. Encouraged by your comments, we have now underlined this fact more boldly in both the abstract and the conclusion sections of the paper. (Track changes version lines 42-48 and 441-457 respectively; clean revised version lines 42-47 and 437-453).*

*The relevant portion of the abstract now reads:*

*In Indonesia, unweighted survey data appeared to substantially over-estimate the health threat posed by substandard or falsified medicines. The types of sampling bias that led to over-representation of poor quality products in our survey are also indicated in other published surveys, possibly exaggerating the scale of the threat to public health posed by substandard and falsified medicines globally. Weighting survey results by sales volume likely improves robustness of estimates of medicine quality measured in field surveys.*

*The relevant portion of the conclusion now reads:*

*Although our study is one of the most comprehensive single-country academic surveys of medicine quality, we found that "headline" figure of 8.2% substandard medicines it yielded likely overestimated both prevalence of poor quality products and their threat to public health. When adjusted for brand volumes, just 0.5% deviated from permitted limits by more than 10%. The overestimate was caused by sampling techniques which over-represented brands with small market share, and medicines bought from unregulated sources. Other published studies do not report relative market share. However, the diversity of brands and outlets they report within small sample sizes suggests that sampling techniques commonly oversample marginal brands which, in our study, were more likely to fail testing. Many also sample from unregulated outlets, where falsified medicines are most commonly found. In our study, we found that informal markets and unregulated internet sources represent only a small proportion of sales, again leading to over-representation of falsified products in our survey compared with the market. This may differ in other markets; we encourage systematic research to quantify the relative size of different market segments in other countries.*

*In common with authors of recent systematic reviews, we believe the aggregation of results across dissimilar studies is misleading.[3,5,6,34] While quality-assurance will differ in countries with different regulatory capacity and medicine import profiles, our study suggests that such aggregations may compound the effect of sampling biases. Together with binary pass/fail reporting of medicines which fail testing only marginally, this has probably led to an overestimate of the potential health threat posed by substandard and falsified medicines globally.*

Otherwise the article is well written and seems to have a unique and detailed methodological approach.

*Thank you.*

Reviewer #2: The paper is a clearly written report of a well-designed study to investigate the scale of falsified medicines in Indonesia, which uses a robust and well developed sampling frame, and a unique weighting procedure to weight by market share, which more accurately captures the real-world impact of falsified medicine.

The paper makes a strong contribution and I don’t have any major suggestions. I would suggest some small edits for clarity:

*Thank you.*

Abstract: ’46.9% lower than the raw estimate’– is this % decline to this level of accuracy and decimal points important for the abstract?

*We think it is important to provide the magnitude, but agree that the decimal point is an unnecessary level of accuracy. We have rounded it to 47%.*

Line 83: “many patients buy medicines outside the public system”.- can you say more about what this might entail and how common is it for consumers to do this?

*Self-treatment is huge! (80%) We have recent statistics and a Statistic Bureau source following line 88-93 track changes version, 86-91 clean version. It now reads:*

*Medicines are provided free to insured patients, but administrative procedures are burdensome and many patients buy medicines outside the public system. In a nationally-representative survey of 345,000 households conducted in 2023, 80% of people who reported any symptoms of illness in the preceding month said they had self-treated. Only 35% of those with symptoms visited a health facility. Medicines accounted for just under a fifth of all out-of-pocket spending on health. Most of this spending was on modern rather than traditional medicines (42% on over-the-counter medicines and 39% on medicines bought with a prescription) acquired directly from health personnel.[15]*

Line 103: ‘chosen based on public health importance’ – you could here reassure the reader that this was done by a committee/consensus of experts or stakeholders.

*In lines 113-117 track changes version, 111-115 clean version, we have itemised some of the of "other stakeholders" mentioned in the text, as well as providing more details of the factors considered in choosing medicines.*

*It now reads: STARmeds medicines were chosen based on public health importance, diversity of suppliers, risk of falsification and feasibility, in consultation with BPOM, the Ministry of Health, the national health insurer, the association of hospitals, and other stakeholders. In determining public health importance we considered the prevalence of the conditions treated by the medicine; the volumes procured in the public system and sold in pharmacies nationally; and the financial burden on the public health insurance system.*

Line 116: ‘selecting target outlets randomly.’: you could explain here that this means across all types, not within types.
Line 119/table 2: it would be good to know within the sample frames, what were the shares of public system/private and other (including medicine shops etc)

*We are sorry that we did not provide enough detail about sampling. We did, in fact, randomise within types. We have provided additional information to clarify, adding also information about numbers of different outlet types as requested. Lines 130-139 track changes version, 128-137 clean version.*

*It now reads:*

*In consultation with experts from Statistics Indonesia, we drew up a sample frame which took into account the relative size of each sampling location, and specified the desired number of sampling venues for each district and outlet type. We then selected target outlets randomly from the list for each location and outlet type, using Stata 17's random generation function.*

*All pharmacies and medicine shops were privately owned; numbers depended on the location. We selected a district public hospital (in most districts there is only one), one private hospital, and one public primary health centre (puskesmas) in each sampling area. All doctors and midwives included were in private practice. We randomly chose a total of five in each location. Further details are provided in S1*

Line 185: what does this n=467 refer to? And does the IQVIA data relate to Indonesia and even areas or Indonesia you sample?

*Sorry this was not clear. The 467 refers to 467 individual products listed by IQVIA with any sales in the Indonesian market for calendar 2022 which fall within the scope of our study. Each is a unique combination of medicine, formulation, dose and brand (or, for unbranded generics, market authorisation holder). We have clarified this in the text in lines 206-209 track changes version, 204-207 clean version. It now reads:*

*For calendar 2022, pharmaceutical data aggregator IQVIA listed non-zero sales volumes in the Indonesian market for 467 separate versions of the medicines and formulations included in the study. Each was a unique combination of medicine, formulation, dose and brand (or, for unbranded generics, market authorisation holder). We used these volumes as the basis for our estimates*.
